# Butylated Hydroxyanisole Blocks the Occurrence of Tumor Associated Macrophages in Tobacco Smoke Carcinogen-Induced Lung Tumorigenesis

**DOI:** 10.3390/cancers5041643

**Published:** 2013-12-04

**Authors:** Yan Zhang, Swati Choksi, Zheng-Gang Liu

**Affiliations:** Cell and Cancer Biology Branch, Center for Cancer Research, National Cancer Institute, National Institutes of Health, Bethesda, MD 20892, USA; E-Mails: zhangy9@mail.nih.gov (Y.Z.); choksis@mail.nih.gov (S.C.)

**Keywords:** tumor associated macrophage, tumorigenesis, lung cancer, NNK, BHA, ROS

## Abstract

Tumor-associated macrophages (TAMs) promote tumorigenesis because of their proangiogenic and immune-suppressive functions. Here, we report that butylated hydroxyanisole (BHA) blocks occurrence of tumor associated macrophages (TAMs) in tobacco smoke carcinogen-induced lung tumorigenesis. Continuous administration of butylated hydroxyanisole (BHA), a ROS inhibitor, before or after NNK treatment significantly blocked tumor development, although less effectively when BHA is administered after NNK treatment. Strikingly, BHA abolished the occurrence of F4/80^+^ macrophages with similar efficiency no matter whether it was administered before or after NNK treatment. Detection of cells from bronchioalveolar lavage fluid (BALF) confirmed that BHA markedly inhibited the accumulation of macrophages while slightly reducing the number of lymphocytes that were induced by NNK. Immunohistological staining showed that BHA specifically abolished the occurrence of CD206^+^ TAMs when it was administered before or after NNK treatment. Western blot analysis of TAMs markers, arginase I and Ym-1, showed that BHA blocked NNK-induced TAMs accumulation. Our study clearly demonstrated that inhibiting the occurrence of TAMs by BHA contributes to the inhibition of tobacco smoke carcinogen-induced tumorigenesis, suggesting ROS inhibitors may serve as a therapeutic target for treating smoke-induced lung cancer.

## 1. Introduction

Lung cancer is the leading cause of cancer-related mortalities in men and women, and despite extensive antismoking campaigns, it still accounts for 15% of all new cancers and 29% of all cancer deaths in the U.S. [1]. Among lung cancers, pulmonary adenocarcinoma is the predominant histological type [1]. Tobacco smoking is the major risk factor, estimated to cause 87% of lung cancer cases in the U.S. [2]. Tobacco smoke (TS) contains 4,000 chemical agents, including over 60 carcinogens. Conversion of these compounds to reactive forms (metabolic activation) results in the formation of DNA adducts that cause many of the genetic changes underlying lung cancer. Among the carcinogens found in cigarette smoke, NNK [4-(methylnitrosamino)-1-(3-pyridyl)-butanone] is one of the most abundant [2], which leads to *K-ras*-activating mutations as early events in the pathway leading to lung adenocarcinoma [3,4]. Interestingly, NNK has been shown to promote the formation of ROS in the lung [5] and this ROS is implicated in the formation and proliferation of tumor cells by promoting DNA damage [6].

Macrophages are the most abundant immune cells involved in tumor development [7]. One previous study showed that the pharmacologic depletion of macrophages in different mouse tumor models significantly reduced tumor angiogenesis and progression, suggesting that macrophages are critical components in the tumor microenvironment for tumor progression [8]. Tumor-associated macrophages (TAMs) are M2-like cells and are responsible for many tumor-promoting activities during tumor initiation, progression and metastasis [9]. TAMs play a major role in suppressing the anti-tumor responses of dendritic cells (DCs), cytotoxic T lymphocytes (CTLs), and natural killer (NK) cells [10,11]. It has been shown that blocking the functions of TAMs inhibits tumorigenesis [12,13]. 

Our previous study found that ROS plays a critical role in the differentiation of alternatively activated macrophages and the occurrence of TAMs [14]. In this study, we examined the effect of blocking ROS on NNK-induced tumorigenesis in A/J mice, which is a widely accepted model of lung tumor. Using the ROS inhibitor, BHA, we examined the occurrence of tumors and TAMs in the lungs of mice treated with NNK. We found that the continuous administration of BHA efficiently blocked the occurrence of TAMs and markedly suppressed tumorigenesis in this NNK-induced lung cancer model.

## 2. Results

### 2.1. BHA Suppresses Tobacco Smoke Carcinogen (NNK)-Induced Lung Tumorigenesis

**Figure 1:**
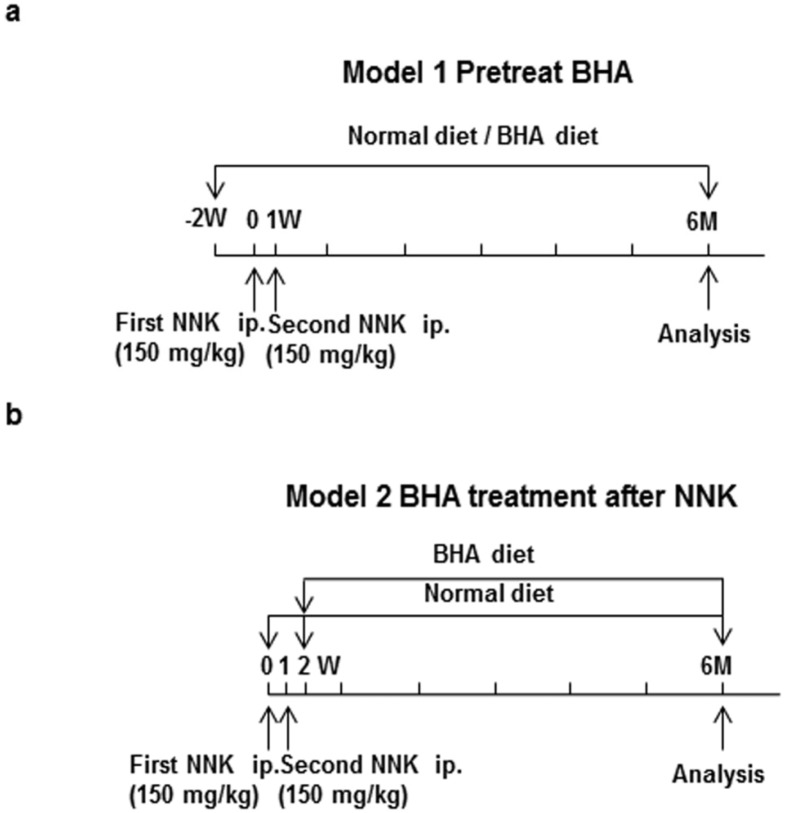
Experimental protocols for promotion of tumor. (**a**) A/J mice were maintained on BHA diet two weeks prior to NNK treatment while control animals were left on normal diet. Tumors were then induced by two separate i.p. injections of NNK, one at day 0 and the second at 1 week; (**b**) A/J mice were injected with NNK at day 0 and week 1, and then maintained on normal or BHA diet starting from week 2. Whole lungs of mice from model 1 and model 2 were collected at 6 months after the first dose of NNK.

To understand the effect of BHA on tobacco smoke-induced lung cancer, we generated two lung tumor models, which were initiated by NNK (Figure 1). In model 1, (Figure 1a), 8-week-old A/J mice were maintained continuously on BHA diet two weeks prior to NNK treatment while control animals were left on normal diet. Tumors were then induced by two separate i.p. injections of NNK, one at day 0 and the second at 1 week. In model 2, (Figure 1b), A/J mice were injected with NNK at day 0 and week 1, and then maintained continuously on BHA diet starting from week 2. Whole lungs of mice from model 1 and model 2 were collected at 6 months after the first dose of NNK (Figure 1). In both models the incidence of tumors was 100% (data not shown). 

**Figure 2:**
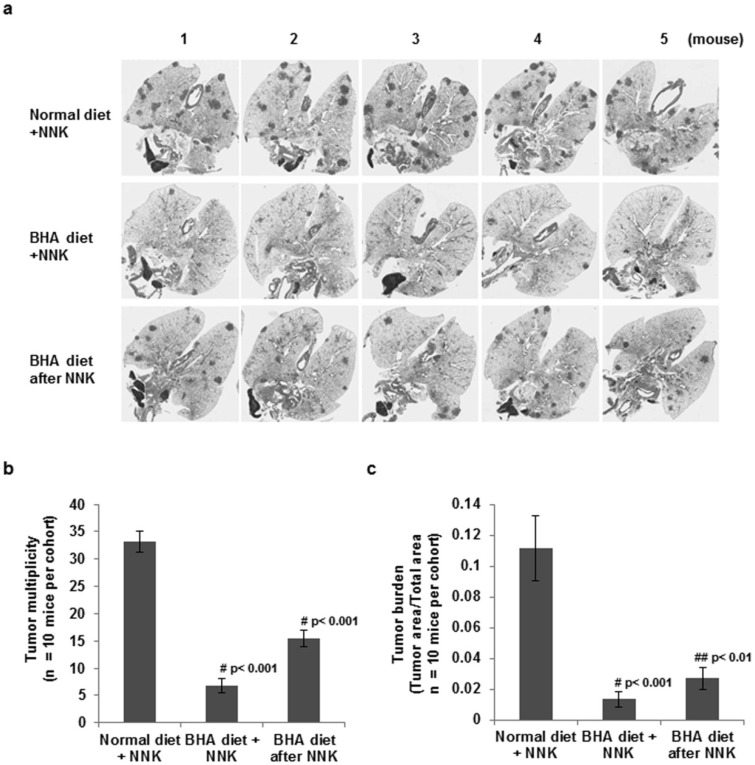
BHA suppresses NNK-induced lung tumorigenesis. (**a**) A/J mice were maintained on normal or BHA diet for 2 weeks prior to NNK injection (Line 1 and 2) or mice on BHA diet 1 week after NNK treatment (Line 3). Lungs were collected at 6 months after NNK treatment and sectioned and stained for H&E. 5 representative H&E images of lungs of mice from each group are shown; (**b**) Lung tumor multiplicity of mice from (**a**) was determined by counting total tumor foci in 5 serial sections at 400 mm intervals; (**c**) Lung tumor burden of mice from (**a**) was shown by the ratio of lung tumor area to total lung are. N = 10; Error Bars: ±SEM.

We examined the lung tumor multiplicity and tumor burden by microscopic examination after serial sectioning of the lungs as previously described [3]. As shown in Figure 2, A/J mice developed large numbers of lung tumors by six months after NNK treatment. The continuous administration of BHA before or after NNK treatment dramatically inhibited the number of tumors in these cancer models as determined by tumor multiplicity after H&E staining (Figure 2a). Statistical analysis showed that administration of BHA either before or after NNK treatment significantly blocked tumor multiplicity (Figure 2b) by 5-fold and tumor burden by 8-fold (Figure 2c) although less effectively when BHA is administered after NNK treatment (2-fold and 4-fold respectively). These results suggest that BHA blocks the progression of NNK-triggered lung cancer. 

### 2.2. BHA Blocks NNK-Induced Occurrence of F4/80^+^ Macrophages in Lungs

**Figure 3:**
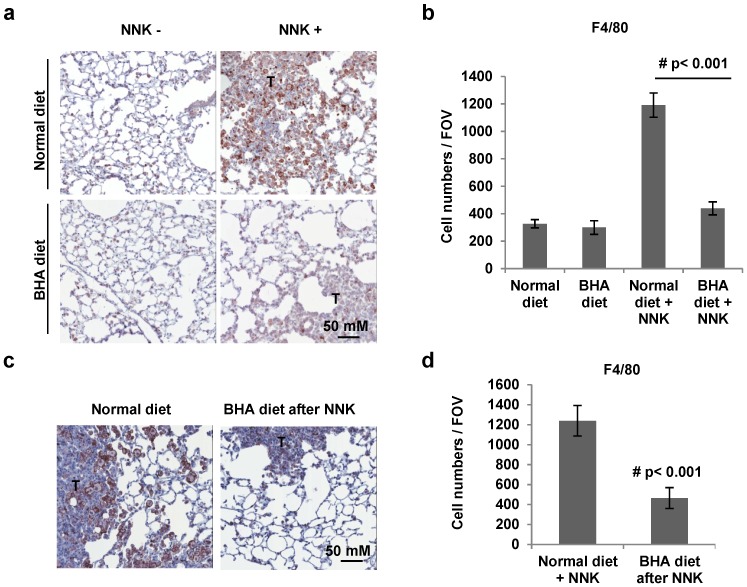
BHA blocks NNK-induced occurrence of F4/80^+^ macrophages in lungs. (**a**) A/J mice were maintained on normal or BHA diet for 2 weeks prior to NNK injection. Lungs were collected at 6 months after NNK treatment and sectioned and immunostained for F4/80. Representative images are shown (T: tumor area); (**b**) Quantitative analysis of (**a**) is shown. The F4/80^+^ cells were evaluated by counting 10 high power fields (20×) per lung section (1 mm)/2 tissue sections/ mouse. *n* = 10 mice; (**c**) A/J mice were maintained on normal or BHA diet 1 week after NNK treatment. Lungs were collected at 6 months after NNK treatment and sectioned and immunostained for F4/80. Representative images are shown (T: tumor area); (**d**) Quantitative analysis of (**c**) is shown.

Since macrophages have been suggested to be critical mediators of tumorigenesis [9], we then tested whether BHA blocks the occurrence of macrophages in the NNK-induced lung tumor models. To quantify the presence of macrophages in tumors, whole lung sections were stained with anti-F4/80 antibody. As shown in Figure 3a, similar background levels of F4/80^+^ macrophages are present in the lungs of control animals on normal and BHA diet. As expected, the numbers of F4/80 positive cells dramatically increased in the lungs of NNK-treated mice compared to that in untreated control mice (Figure 3a). Interestingly, the administration of BHA before NNK treatment strikingly blocked the increase of F4/80^+^ macrophages in the tumor sections of the lungs (Figure 3a). Similar results were seen in mice with BHA administered after NNK treatment (Figure 3c). Statistical analysis showed that administration of BHA either before or after NNK treatment almost completely blocked the increase in the occurrence of F4/80^+^ macrophages in the lungs (Figure 3b,d).

### 2.3. BHA Blocks NNK-Induced Increase of Macrophages in BALF

**Figure 4:**
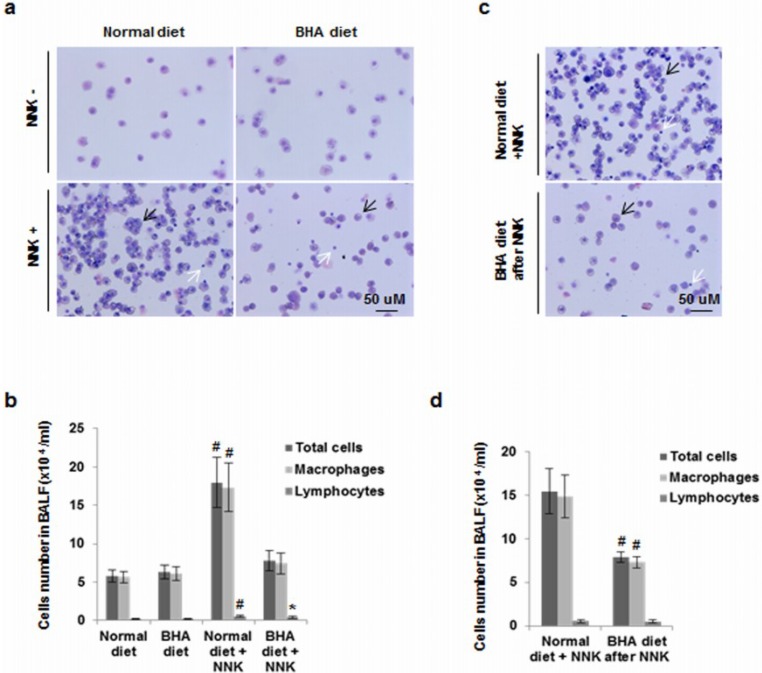
BHA blocks NNK-induced increase of macrophages in BALF. (**a**) A/J mice were maintained on normal or BHA diet for 2 weeks prior to NNK injection. BALF cells were prepared and stained with Wright and Giemsa after two weeks after NNK treatment. Representative images are shown; (**b**) Quantitative analysis of (**a**) is shown. The percentages of leukocyte types in BALF were determined by counting 400 leukocytes in a randomly selected portion of the slide under light microscopy; (**c**) A/J mice were maintained on normal or BHA diet 1 week after NNK treatment. Representative images of BALF cells collected 2 weeks after the last NNK dose. Represetitive Wright-Giemsa stainings are shown; (**d**) Quantitative analysis of (**c**) is shown. Black arrow: macrophage; White arrow: lymphocytes.

To confirm that BHA targets macrophage accumulation in the lungs during development, we examined the numbers of macrophages in bronchoalveolar lavage fluid (BALF) in our animal lung tumor models. BALF was collected at 2 weeks after NNK administration and the total cell number and numbers of various leukocyte types in BALF were quantified. Both macrophages and lymphocytes were significantly increased after NNK exposure (Figure 4a,b). Mice maintained on a BHA diet before NNK treatment showed impaired recruitment of NNK-induced cells to BALF. The numbers of macrophages but not lymphocytes were significantly reduced (Figure 4a,b). Mice maintained on a BHA diet after NNK treatment also had decreased number of NNK-induced macrophages found in the BALF (Figure 4c,d).

### 2.4. BHA Specifically Blocks the NNK-Induced Occurrence of TAMs in Lungs

**Figure 5:**
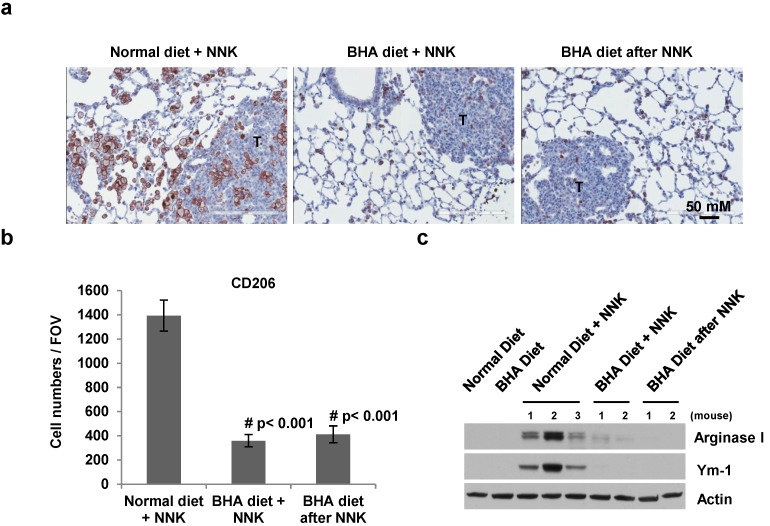
BHA specifically blocks NNK-induced occurrence of TAMs in lungs. (**a**) A/J mice were maintained on normal or BHA diet for 2 weeks prior to NNK injection or BHA diet 1 week after NNK treatment. Lungs were collected at 6 months after NNK treatment and sectioned and immunostained for CD206. Representative images are shown (T: tumor area); (**b**) Quantitative analysis of (**a**) is shown. The CD206^+^ cells were evaluated by counting 10 high power fields (20×) per lung section (1 mm)/2 tissue sections/mouse. *n* = 10 mice; (**c**) Lung tissues from (**a**) were analyzed by western blotting with anti-arginase I, anti-Ym-1 and anti-actin antibodies. Representative images are shown.

Since most macrophages in the tumor microenvironment are TAMs, we then tested whether BHA specifically blocks the occurrence of TAMs in the lung tumor models. CD206 was used as the specific marker for TAMs [15]. As shown in Figure 5a,b, the numbers of CD206 positive cells are dramatically increased, especially in the areas around the tumors, in the lungs of NNK-treated mice. Administration of BHA before NNK treatment markedly blocked the increase of CD206^+^ macrophages in the lungs (Figure 5a,b). Similar results were seen in mice administrated with BHA after NNK treatment (Figure 5a,b). 

To verify that the increased macrophages in NNK-treated mice are mostly TAMs, we examined the expression of TAMs markers Arginase I and Ym-1 in these lung samples by western blotting [15]. As shown in Figure 5c, very low or no expression of Arginase I and Ym-1 was detected in the lungs of control mice on normal or BHA diet. However, the expression levels of Arginase I and Ym-1 were dramatically increased in the lungs of NNK-treated mice. Importantly, this elevated expression of Arginase I and Ym-1 was abolished when BHA was continuously administered to NNK-treated mice either before or after NNK treatment (Figure 5c). These results indicated that BHA specifically blocks the occurrence of TAMs in the NNK-induced lung cancer model. 

## 3. Discussion

Although substantial epidemiological evidence indicates an association between pulmonary inflammation and tobacco smoke-induced lung cancer, a direct causal interaction between which specific-type of inflammatory infiltrates results in tumor initiation and the molecular events underlying inflammation-related lung tumorigenesis are not well known [16]. Our previous study found that ROS plays a critical role in the differentiation of alternatively activated macrophages and TAMs [14]. The continuous administration of the ROS inhibitor, BHA, efficiently blocked the occurrence of TAMs and markedly suppressed tumorigenesis in urethane-induced lung cancer and *K-ras^LA2^*-induced lung cancer [14]. Since tobacco smoking is the major risk factor of lung cancer (Hecht, 2002), here we explored the role of BHA in tobacco smoke carcinogen-induced lung tumorigenesis. 

NNK is the most abundant carcinogen found in cigarette smoke (Hecht, 2002), we therefore used NNK as the chemical to induce lung tumor. Similar to other lung cancer models, the inflammatory microenvironments including macrophages have been reported to play a critical role in promoting lung tumorigenesis in NNK-induced lung cancer model [17]. In addition, *K-ras* mutation in codon 12 of the A/J mice is most commonly detected in preneoplastic hyperplasias or adenocarcinoma induced by NNK treatment [18], which results in a milieu of downstream changes leading to tumorigenesis. NNK induced overexpression of oncogenic *K-ras* leads to the activation of the transcription factor, NF-κB, the enhancement of inflammatory responses and the development of lung adenocarcinomas [19]. Our previous study showed that blocking TAMs leads to the suppression of tumorigenesis in oncogene-driven *K-ras^LA2^*-induced lung cancer, suggesting that TAMs may play an important role in NNK-induced lung tumorigenesis. Chemicals targeting TAMs could prove to be successful for the treatment of tobacco smoke-induced lung cancer.

As expected, we found that continuous administration of BHA blocked tumor development in NNK-induced lung cancer model. Previous studies have looked at the effect of antioxidants, including BHA, on NNK-induced tumorigenesis [20] and have found that it affects tumorigenesis. However, the mechanisms of how BHA effect on tumorigenesis is still unclear. Our study examined the continuous administration of BHA and found that it has a protective effect against NNK-induced lung tumorigenesis in A/J mice which is achieved by specifically blocking the occurrence of TAMs in tumors induced by NNK. Our study suggests the possibility of BHA as an inhibitor of tobacco smoke-induced lung cancer development may be achieved by blocking the accumulation of TAMs. Many factors have been shown to affect the accumulation of TAMs, such as macrophage-colony stimulating factor (M-CSF) which induces monocyte to M2 like macrophage or TAM differentiation [15], IL4 or IL10 which works on the polarization of TAMs, and some chemokines that are specific for M2 macrophages or TAMs accumulations [15]. Our previous study showed that BHA specifically affects M2 macrophages or TAMs accumulation triggered by these factors. BHA blocks macrophage differentiation and this is overcome when cells are polarized to classically activated (M1), but not M2 macrophages. Since TAMs are M2-like macropahges BHA specifically targets TAMs as seen by the loss of Arg and Ym-1 expressions in mouse macrophages. This study supports our finding that BHA effects tobacco smoke-induced lung tumorigenesis by targeting TAMs [14].

Since oxygen free radicals and other oxidative agents produced during the inflammatory response to NNK could lead to DNA damage, some of which could initiate lung tumorigenesis, it is possible that BHA may directly block the oxidative DNA damage that contributes to the *K-ras* mutations. However, we did not observe a difference in tumor incidence (data not shown). Our previous data suggested that BHA has no effect on monocyte migration or the proliferation of tumor cells in a NUDE mouse model depleted of macrophages [14], therefore it is most likely that the BHA suppressed NNK-induced tumor development by specifically affecting the TAMs.

## 4. Materials and Methods

### 4.1. Animals

A/J mice were from the Jackson Laboratory (Bar Harbor, ME, USA). Mice were maintained under pathogen-free conditions, and experimental protocols were approved by NCI, following NIH guidelines.

### 4.2. Reagents and Antibody

BHA was obtained from Sigma (St. Louis, MO, USA). NNK was obtained from Toronto Research Chemicals (Toronto, ON, Canada). Anti-Ym-1 from STEMCELL Technologies (Vancouver, BC, Canada); anti-Arginase I from Santa Cruz (Dallas, TX, USA); anti-CD206 from Hycult Biotech (Plymouth Meeting, PA, USA); and anti-F4/80 from eBiosciences (San Diego, CA, USA). 

### 4.3. Western Blot Analysis

Cells were collected and lysed in M2 buffer (20 mM Tris at pH 7, 0.5% NP-40, 250 mM NaCl, 3 mM EDTA, 3 mM EGTA, 2 mM DTT, 0.5 mM PMSF, 20 mM β-glycerol phosphate, 1 mM sodium vanadate, and 1 mg/mL leupeptin). Cell lysates were separated by SDS-PAGE and analyzed by immunoblot. The proteins were visualized by enhanced chemiluminescence (ECL, Pierce, Rockford, IL, USA).

### 4.4. NNK-Induced Lung Tumor Models

Eight-week old female A/J mice that were weight and age matched were used for experiments. Tumors were induced by two i.p. injection of 150 mg/kg NNK. Animals were then divided into normal NIH-31 chow or NIH-31 chow with 7.5 g/Kg BHA. After 6 months lungs were excised and evaluated for tumors.

### 4.5. Evaluation of Lung Tumors

For determining tumor multiplicity and tumor burden, whole lungs were inflated with and fixed in 4% paraformaldehyde for 24 h. Lungs were paraffin-embedded and serial sections at 400 microns were histologically examined with hematoxylin and eosin (H&E) stain. For quantitation of lung tumor multiplicity, tumor numbers of 5 serial sections per lung were counted and totaled. Tumor burden were shown as the ratio of tumor area to total lung area on 5 sections taken every 400 microns following staining with H&E by using imagescope. 10 lungs were analyzed for each cohort indicated.

### 4.6. Immunohistochemical Analysis

Paraffin-embedded slides were deparaffinized and antigens were unmasked by autoclaving at 121 °C for 10 min in Sodium Citrate (pH 6.0) buffer. Slides were incubated with primary antibody (anti-F4/80 and anti-CD206) in 4 °C overnight. Signals were detected with VECTASTIN ABC Elite kit (Vector Laboratories, Burlingame, CA, USA) and DAB Substrate Kit (Vector Laboratories). Quantitative analysis of positive cells was performed by counting cells in ten high-power fields (20×) per two tissue sections from 6 to 10 mice per group.

### 4.7. BALF Leukocyte Counts

BALF was withdrawn after instillation of 800 μL sterile PBS through the trachea, and cytospin preparations of BALF cells were prepared using Shandon Cytospin centrifuge (Thermo Electron Corporation, Waltham, MA, USA). BALF cells were visualized by Wright-Giemsa staining and percentages of leukocyte types were determined by counting 400 leukocytes in a randomly selected portion of the slide under light microscopy. BALF total leukocyte counts were performed using a hemocytometer. 

### 4.8. Statistical Analysis

Statistical analyses were performed using GraphPad Prism and/or Aperio ImageScope Software. Two group comparisons were performed using Student’s *t* test. All p values less than 0.05 were considered statistically significant.

## 5. Conclusions

In summary, our data clearly demonstrated that inhibiting the occurrence of TAMs by BHA contributes to its inhibition of tumorigenesis. Considering the importance of TAMs, our study suggested that the continuous administration of BHA or other antioxidants for the targeting of TAMs may be a potentially effective method for tobacco smoke-induced cancer treatment. Although BHA could have multiple targets during lung tumor development, as a cancer prevention and therapeutic agent, BHA may be more potent if it inhibits multiple components of tumorigenesis. Our previous study demonstrated that BHA has no effect on the classic inflammatory macrophages involved in the inflammation responses or on local macrophages in non-cancer mice [14]. In addition, we found that continuous BHA treatment is not toxic to mice as we did not notice any developmental abnormalities or diseases including cancer in mice on BHA diet for up to two years (data not shown), suggesting BHA could be useful as a powerful therapy against tobacco smoke-induced lung cancer.
